# Mapping obesity and diabetes’ representation on Twitter: the case of Italy

**DOI:** 10.3389/fsoc.2023.1155849

**Published:** 2023-06-15

**Authors:** Francesca Romana Lenzi, Ferdinando Iazzetta

**Affiliations:** ^1^Laboratory of Psychology and Social Processes in Sport, Department of Movement, Human and Health Sciences, University of Rome “Foro Italico”, Rome, Italy; ^2^Department of History, Anthropology, Religions, Performing Arts - Ph.D. in History and Cultures of Europe, University of Rome “La Sapienza”, Rome, Italy

**Keywords:** health, diabetes, obesity, twitter, sentiment (SEN) analysis, content analaysis, T-LAB linguistic software

## Abstract

One of the main functions of public health is to monitor population health to identify health problems and priorities. Social media is increasingly being used to promote it. This study aims to investigate the field of diabetes and obesity and related tweets in the context of health and disease. The database extracted using academic APIs (Application Programming Interfaces) allowed the study to be run with content analysis and sentiment analysis techniques. These two analysis techniques are some of the tools of choice for the intended objectives. Content analysis facilitated the representation of a concept and a connection between two or more concepts, such as diabetes and obesity, on a purely text-based social platform such as Twitter. Sentiment analysis therefore allowed us to explore the emotional aspect related to the collected data related to the representation of such concepts. The results show a variety of representations connected to the two concepts and their correlations. From them it was possible to produce some clusters of elementary contexts and structure narrative and representational dimensions of the investigated concepts. The use of sentiment analysis and content analysis and cluster output to represent complex contexts such as diabetes and obesity for a social media community could increase knowledge of how virtual platforms impact fragile categories, facilitating concrete spillovers into public health strategies.

## 1. Introduction

Health is identified and recognized as reflecting the state of wellbeing of an individual and a society. Since WHO’s first definition in 1946, there have been several evolutions of the concept of health itself over time. In the new information age (Castells, 1996), the concept of digital health, understood as the use of information and communication technologies in medicine to manage disease and health risks and to promote wellbeing (Kostkova, 2015; Mathews et al., 2019; Rieke et al., 2020), is gaining momentum.

The web and social media have become very important venues for discussing health issues and for providing ways to seek out and interact with communities of patients with similar conditions or professionals, not only to gather information but also to discuss problems and feelings, ask for help, support others, and get support from others (Farmer et al., 2009; Hawn, 2009). Actors inhabiting the digital public sphere also contribute to the public discourse on health and wellbeing through the production of big data by means of different modalities. User-generated health data are naturally occurring digital traces (Peng et al., 2019) and can be generated by social networks (Ayers et al., 2016), wearable devices and health apps (Casselman et al., 2017), and search engines (Mavragani et al., 2018). The proliferation of user-generated content is an element that generates an impact on the production, circulation, and consumption of health-related news and ensures a vibrant public sphere on the topic (Hodgetts et al., 2008). Today, it is critical to identify how the new datasets produced in health terms can be used to assess Social Determinants of Health (SDH)^1^ understood as nonmedical factors that influence health outcomes and include the conditions in which people were born, grow, work, live, and get older and include what shapes the conditions of daily life (WHO, 2021).^2^

In this scenario, health technology holds enormous promise for building digital health literacy skills and improving health outcomes for patients with chronic diseases. Contemporary societies have undergone an epidemiological transition (Omran, 1971) that has seen diseases with an infectious prevalence turn into chronic degenerative diseases over the years.

The growth of social media has provided a research opportunity to track public behavior, information, and opinions on common health problems. Today, social media can provide timely public health information, such as tracking or predicting the spread of COVID-19. Several studies show how data extracted from social networks have been used to study the evaluation of vaccination campaign sentiments or mental health problems during the COVID-19 pandemic. Several authors have applied machine learning models to monitor the level of stress, anxiety, and loneliness during the pandemic using Twitter data (Guntuku et al., 2020; Zhang et al., 2020).

Nowadays, health information from social networks’ traditional sources has the potential to change patterns of health inequalities and access to healthcare (Griffiths et al., 2012) by providing a unique opportunity to understand users’ opinions concerning common health problems (Mejova et al., 2015).

Therefore, it is critical to consider social media both as an effective way to engage the public and communicate key “public health” messages and as a valuable data source for detecting or predicting diseases or conditions. Data sources that, if harnessed appropriately, can provide local and timely information about diseases and related events, and are interpreted as the concept of digital epidemiology.

Digital epidemiology can be broadly defined as epidemiology that uses digital data that are not properly generated for the main purpose of epidemiological studies. It involves the treatment of digital methods from the collection stage to the analysis stage (Eckhoff and Tatem, 2015; Salathé, 2018). The goal of digital epidemiology is identical to that of traditional epidemiology, namely, the study of the various factors that influence the occurrence, distribution, prevention, and control of diseases, injuries, and other health-related events in a defined population. The goal of epidemiological studies is not simply to identify the causes of a disease but to apply the findings to prevention and health promotion (WHO, 2004).^3^ In particular, epidemiological studies such as sociological research—conducted within health services—are efficient to synthesize the description of the temporal and geographic distribution of diseases in communities, the relationship between specialized knowledge (medical, psychological, psychiatric, and social), and the health status of the population, besides the evaluation of the therapeutic effectiveness produced in public and private healthcare.

Relevant literature indicates that ubiquitous access to social media can help promote healthier lifestyles (Jiang and Yang, 2017). Risk factors, such as drug abuse, smoking, poor diet and exercise, and associated diseases, are often clustered in the population. A better understanding of social media and related health data will help expand the utility of social media in public health.

## 2. Materials and methods

### 2.1. Research framework

The burden of disease in economic, political, social, and, above all, human terms is intolerably high in most of the world, specifically when considering the poorest areas, but even when considering the impact of toxic lifestyles and the environment that affect human beings without differentiating their socioeconomic conditions. The pandemic showed us that this fragile equilibrium cannot face an emergency like COVID-19 because it does not respond to an acceptable normality in terms of sanitary systems’ capacity to face the degeneration of human health conditions. If we can live longer, it means that we are older and, consequently, more vulnerable than ever (Napier, 2014; Lenzi and Vaccaro, 2019).

Current epidemiological challenges include reducing the prevalence of communicable and non-communicable diseases (NCDs). Diabetes—in this case, with a specific focus on type 2 diabetes—and obesity are two diseases that are often interrelated.

According to the World Health Organization, global obesity has nearly tripled since 1975 (Vespasiani et al., 2005; Leitner et al., 2017; Lenzi and Filardi, 2022; Veit et al., 2022). In 2016, more than 1.9 billion adults were overweight; of these, more than 650 million were obese.

Overweight and obesity have a multiplicative and dangerous relationship: worldwide, obesity and type 2 diabetes are on the increase and are among the chronic degenerative diseases that most affect people’s health. In Italy, according to ISTAT^4^ data, for the 4 years (2017–2020), the prevalence of diabetes is estimated at 5.9%, corresponding to more than 3.5 million people, with a slowly increasing trend in recent years. Today, for people at risk of obesity and type 2 diabetes, it is possible to talk about prevention, drastically reducing the chance of getting sick and avoiding risk factors such as unhealthy lifestyles, obesity, and sedentary lifestyles. In Italy, nearly 22 million people are overweight, 6 million people are obese, and 3.5 million people have diabetes.

Social media provides an open forum for communication among individuals, and Twitter, with its 280-character tweet, has become a popular platform for conversations about health conditions, diseases, and medications (Prieto et al., 2014) and is an effective information channel for practitioners to provide relevant information (Pulman, 2009).

### 2.2. Research object

Considering the perspective of studies between sociology and health, the objective of the present study was to explore the themes, debate, and sentiment of tweets mentioning “obesity” and “diabetes” to analyze the semantic content and conversations taking place on this topic. In this study, therefore, the characteristics of the relationship between diabetes and obesity-related non-symmetrical but supportive concepts of disease/health are identified. The research questions from which the research moves are as follows: What themes prevail, in salient terms, in the conversations produced on Twitter? What kind of narratives do diabetes and obesity have in common?

Content posted on social media has an impact on people and their decision-making. Knowing the sentiment toward diabetes and obesity is crucial to understand the impact such information might have on people with this health condition and their family members. For this purpose, we have selected the social platform Twitter as the context unit. The empirical basis is the extraction of tweets in the Italian language using API (Application Programming Interface) respecting the two queries “Obesity” and “Diabetes” connected to keywords such as “health” and “disease.” The data automatically extracted and collected in natively digital matrices (Rogers, 2013; Caliandro, 2018) cover an observation period of 4 years, from 1 January 2019 to 31 December 2022. The variables included are dates, full tweet corpus, but also likes and retweets (RT) (dichotomous YES/NO).

For this purpose, text data mining operations using automatic text analysis and content analysis techniques are needed (Losito, 1996; Bolasco, 1999; Amaturo and Punziano, 2013). It is precisely through content analysis that it is possible to identify the themes through which communication is organized and the analysis of which words co-occur in the text.

There are some pitfalls in social media mining. First, text data can be difficult to classify and interpret because the data collected may not provide enough information and meaning to facilitate automatic classification. In addition, while coding for geographic origins can resolve some limitations, not all profile accounts on social networking sites contain geographic information, and visible geographic information cannot be easily verified. To avoid the risk of missing relevant information, other strategies are used that better fit the exploratory nature of the objective. We have extracted all tweets with the keywords “diabetes and obesity” associated with keywords such as health or disease. Next, the relevance exclusion methodology includes the elimination of retweets without additional information (comments), returning a set of 22,354 tweets from the first extraction (Table 1).

**Table 1:** Year for total tweet.

Year	Total tweet
2019	7,284
2020	4,567
2021	4,832
2022	5,671
TOTAL	22,354

### 2.3. A two-phase methodological architecture

The analysis architecture involves two phases. The first part of corpus processing involves an automatic phase carried out by T-Lab, which performs normalization, polythene selection, vocabulary construction, and corpus segmentation according to punctuation usage, number of characters, and statistical criteria (Lancia, 2012). Finally, lemmatization is carried out, which will be further refined in the keyword selection phase. Following this automatic phase, some quantitative characteristics of the corpus are evaluated to determine whether it is possible to process the data statistically (Bolasco, 1999; Giuliano and La Rocca, 2010). The elementary context analysis examines common themes and topic patterns to understand the prevailing factors and allows the construction of a corpus content map according to the co-occurrence of the selected keywords. The T-Lab software enables the construction and exploration of a representation of corpus content through a few thematic clusters consisting of elementary contexts described by lexical units. The chosen analysis procedure is the unsupervised clustering method and involves grouping the lexical units in the keyword list under the same root and selecting the keywords according to some exclusion criteria by eliminating words such as:

1) the words that belong to the high-frequency rank (drop point), since they are taken for granted in the context of the treated topic (Bolasco, 1999);
2) words that belong to the low-frequency rank because, by going to specify, they make noise and do not allow us to see regularities.

This procedure involves the use of the cosine measure and clustering of context units using the bisecting K-means method, the construction of a table of lexical unit contingency by cluster, the chi-square test applied to all cluster and lexical unit crossings, and the analysis of lexical unit contingency table matches by cluster (Benzécri, 1984). Then in the second phase, we use natural language processing (NLP) techniques and qualitative sociolinguistic analysis by calculating the sentiment scores of tweets (Gabarron et al., 2019).

The segmentation of the text into elementary contexts is classified by fragments, i.e., elementary contexts of comparable length consisting of one or more utterances.

Among computational methods for analyzing tweets, computational linguistics is a well-known approach developed to obtain information about a population, track health issues, and discover new knowledge (Paul and Dredze, 2011; Harris et al., 2014).

The technique used evaluates and combines two types of algorithms to improve the quality of the results: the Bert-Italian-cased sentiment model and the Van ADER, an acronym for Valence Aware Dictionary and Sentiment Reasoner (Gilbert, 2016). In the second phase, some preprocessing steps are also performed using the natural language processing library to segment the entire corpus with a Bert model that follows a dictionary specific to the Italian language.

The Bert model uses the sentiment intensity analyzer (SIA) with a deep, unsupervised, and, therefore, pre-trained two-way linguistic representation model to analyze sentiment in text data. The unsupervised learning model suggests that the system receives a set of unlabeled data in the training phase that will be classified based on common features (Di Giovanni and Brambilla, 2021).

The first line of the code imports SIA and vader_lexicon (VADER) (a lexicon of words and their sentiment scores) creating an empty list to store sentiment scores, sentiment intensity, and neutrality percentage. The model loops over each case in the “tweet” column of the array, passing each text to the polarity_scores() method of SIA. VADER provides a Python library that can be integrated into any project, and in addition, the model can be fine-tuned to fit specific use cases, such as analyzing domain-specific texts, which in our case allows for more accurate results. This model produces sentiment scores ranging from −1 to 1, where −1 is the most negative, 0 is neutral, and 1 is the most positive. The code then adds the composite score to the sentiment_score list, the neutral percentage to the neutral list, and the sentiment magnitude to the sentiment_magnitude list. The model returns the following three new variables: sentiment_score, neutral, and sentiment_magnitude by returning the compound score, neutral percentage, and sentiment magnitude for each case in the ‘tweet’ column (Jiang and Yang, 2017).

Positive sentiment: compound value >0.001, assigned score = 1.
Neutral sentiment: (compound value > −0.001) and (compound value <0.001), assigned score = 0.
Negative sentiment: compound value < −0.001, assigned score = −1.

## 3. Results

The analyzed corpus appears to be rich from a lexicometric point of view, as the TTR ratio (0.065) is <0.1 and Hapax/Type (0.462) is <0.50. The analysis procedure for partitioning produces four clusters that are organized within a factorial space of *n* = 3 factors (three latent dimensions that explain the maximum total variance of the data).

By applying this procedure to the groups of lemmas extracted for the polarities of each of the three extracted factors (automatically extracted as a result of the algorithm applied by the software), the latent meaning of the representations that emerge for each factor may be reconstructed. Therefore, we report the most significant lemmas extracted for each factor (Table 2).

**Table 2:** Factor summary (list of the top 15 lexical units sorted by absolute contribution and percentage of inertia explained by factors).

Factor 1–44.09% Research		Factor 2–30.49% Pathology		Factor 3–25.40% Prevention	
Pole (+)	Pole (−)	Pole (+)	Pole (−)	Pole (+)	Pole (−)
New	Rischio	Diet	Pathology	Type	Medicine
Type2	Problemi	Mangiare	Problemi	Day	Care
drug	Pear	Covid	Helping	World	New
telemedicine	Eat	Factor	Food	Italy	Prevention
worldwide	Pathology	Hypertension	Alimentary	Drug	PNRR
prevention	Overweight	Die	Agile	Patient	Studio
patients	Increase	Diabetes mellitus	Profession	Committee the invisible patients (Comitato i malati invisibili)	Health
Italy	Understand	Sugar	Psychoanalytic	Invisible patients	Insulin
screening	Factor	Comorbidities	Mediterranean	Committee	Healthcare
Study	Diet	Person	Psychotherapist	Milion	innovation
Healthcare	Grease	Age	Aimed at	Committee onlus	Therapy
Medicine	Disregard	Serious	Blood glucose	TG24	Congress
Therapy	Text	Cause	Psychiatrist	November	Research
PNRR	Flexible	Death	Blood	Sky	City
Care	Cardiovascular	Vaccinate	Reading	Diabetics	Cell

**Figure 1:**
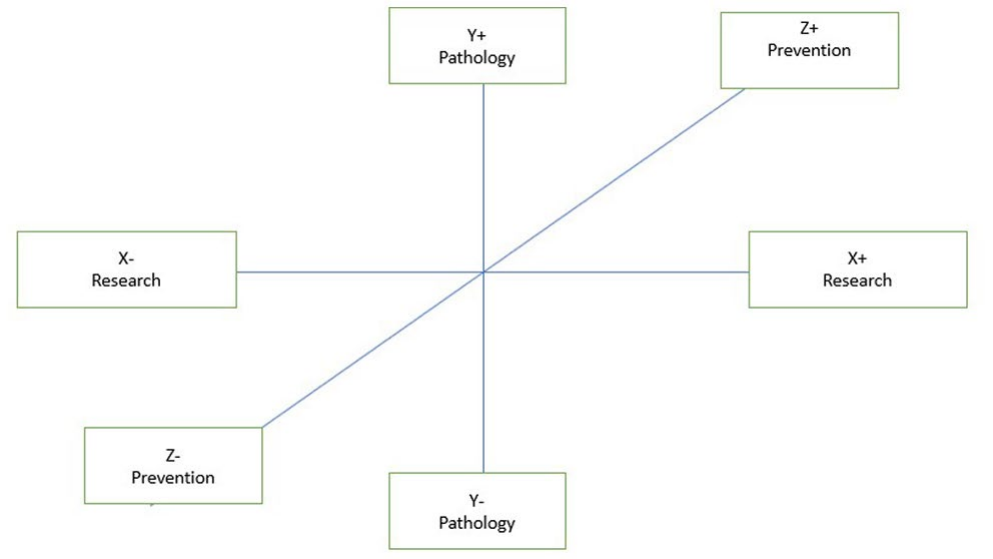
Interpretation factor axes.

The three factors are shifted reciprocally on the x-axis and y-axis to capture the different nuances that can be observed in “photographs” portraying the same object, taken, however, from different angles, according to the metaphor proposed by Lancia (2012) (Figure 1).

As the repetition of similar words observed by scrolling through the individual clusters allows the GAP index not to be overwritten but at the same time to follow a more symmetrical logic, four clusters are chosen. The words within each axis are interpreted to recognize the dimensions that identify the characterizing cultural space.

The number of elementary contexts classified is 22,354 (=96.9%; out of a total of 23,069). Of interest for interpretation, it can be seen immediately that the clusters with the most weight are number 2 and number 3, with 31 and 28% of the elementary contexts classified, respectively, as shown in Table 3.

**Table 3:** Clusters by classified elementary contexts.

CLUSTER 1	4,231	18.93%
CLUSTER 2	6,998	31.31%
CLUSTER 3	6,400	28.63%
CLUSTER 4	4,725	21.14%

**Figure 2:**
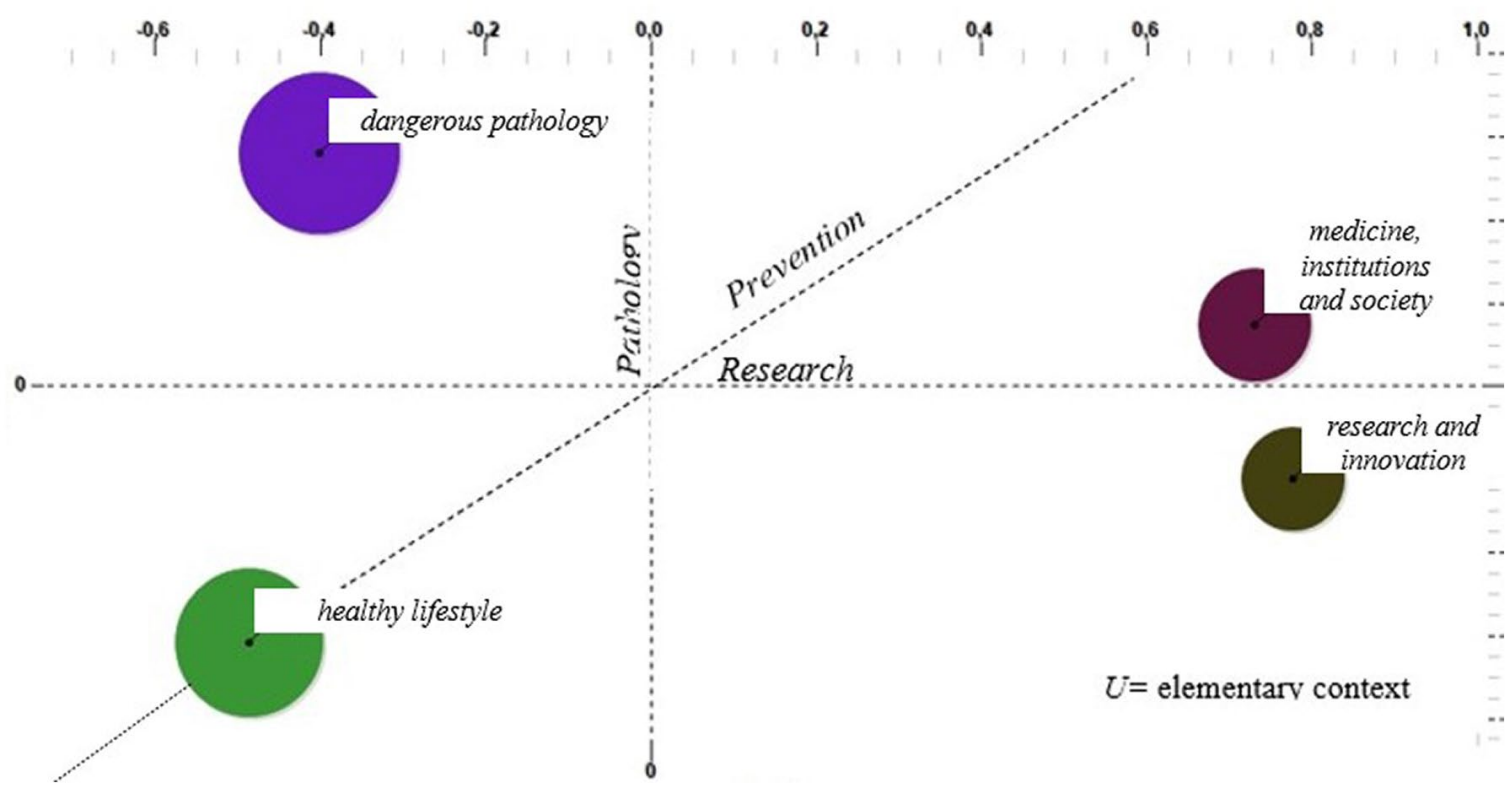
Factorial plan and clusters: Processing with T-Lab.

The clustering procedure involves the use of cosine measurement and context unit clustering using the k-means method, i.e., the construction of a contingency table per cluster. The clusters have also been represented on a factorial plan to show proximity or dispersion related to the elementary context in diabetes and obesity’s representation and narrativity, as shown in Figure 2.

**Figure 3:**
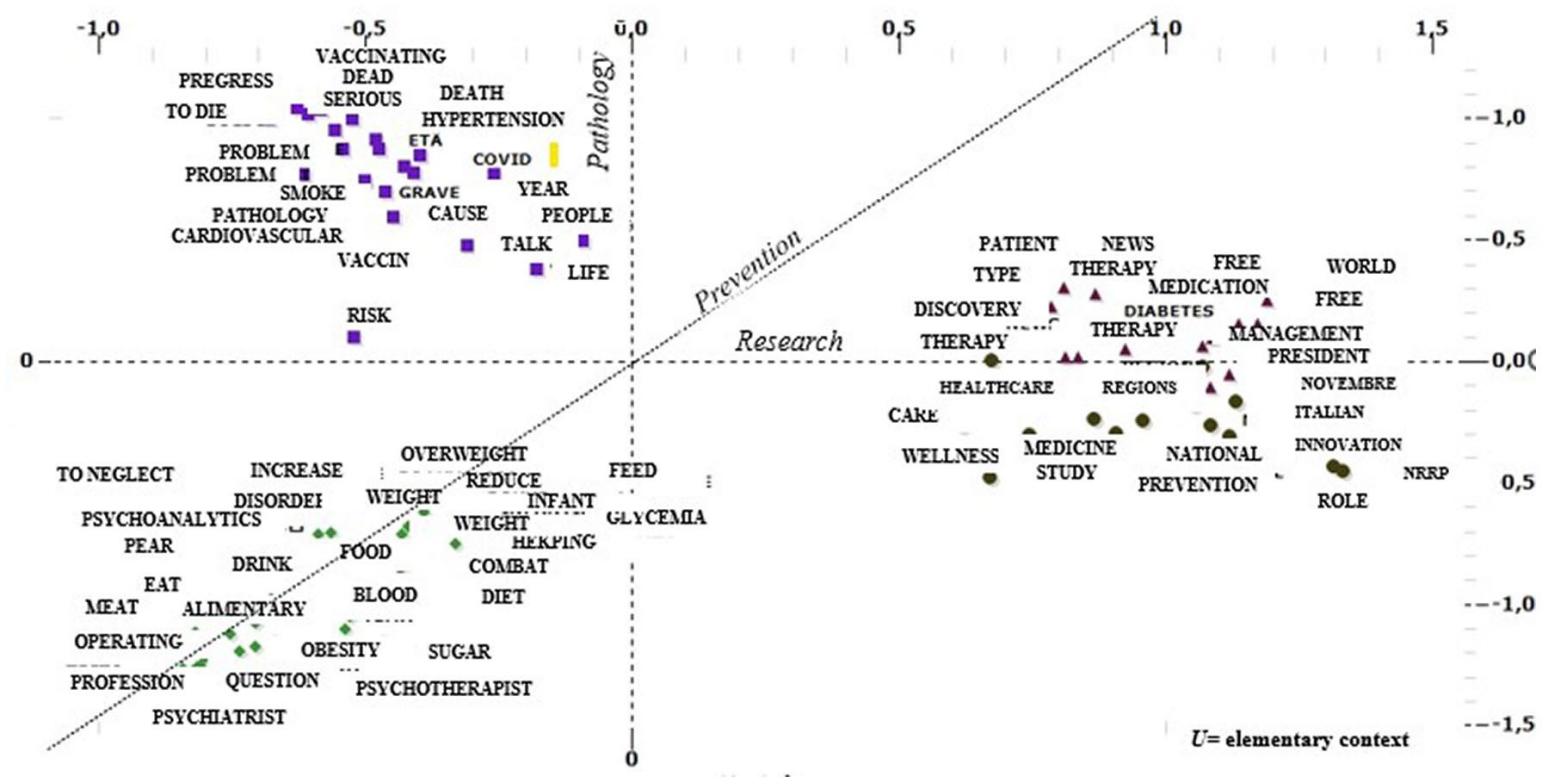
Factorial plan on lemmas and clusters: Processing with T-Lab.

The clusters are the product of grouping lemmas that can refer to the same representative matrix, as shown in Figure 3.

T-Lab has allowed the disclosure of lemmas after their clustering. For example, Table 4 shows the first 15 characteristic lemmas for each of the four clusters: The lemmas are extrapolated with the Who square test, a statistical test designed to check whether the frequency values obtained by survey and recorded in some double-entry tables are significantly different from the “theoretical” (or expected) values (Corbetta, 2014).

**Table 4:** The first 15 lemmas sorted by co-occurrences and CHI^2^.

Cluster 1				Cluster 2				Cluster 3				Cluster 4			
LEMMAS-VARIABLES	IN CLU	IN TOT	CHI^2^	LEMMAS-VARIABLES	IN CLU	IN TOT	CHI^2^	LEMMAS-VARIABLES	IN CLU	IN TOT	CHI^2^	LEMMAS-VARIABLES	IN CLU	IN TOT	CHI^2^
New	736	1,098	1831,513	Pathology	1,049	1,282	1,316,782	Diet	869	1,131	1,258,387	Type2	1,284	1820	3,130,012
Prevention	539	836	1,251,77	Problem	922	1,138	1,129,009	Eat	517	593	974,051	Day	625	669	2,388,974
Medicine	382	501	1,174,216	Covid	851	1,148	829,756	Alimentary	383	417	798,578	World	576	605	2,264,734
Study	512	893	959,77	Factor	627	751	823,734	Sugar	451	558	726,803	Italy	625	867	1,574,075
Care	486	844	918,296	Die	418	458	671,596	Overweight	783	1,252	684,386	Drug	621	887	1,486,645
PNRR	181	187	799,152	Hypertension	621	811	658,568	Agile	267	267	652,28	Patient	607	931	1,279,264
Healthcare	347	552	769,363	Dead	386	423	619,874	Profession	267	268	647,856	Diabetes	4,309	15,528	800,685
Health	342	559	722,1	Severe	367	420	535,065	Text	266	267	645,409	Invisible patients committee	98	98	413,504
Innovation	140	148	597,684	Years	752	1,150	508,799	Psychoanalytic	264	264	644,941	Invisible patients	97	97	409,282
Insulin	289	495	559,484	Cardiovascular	476	710	347,244	Operating	266	276	607,167	Comitatoimi	95	95	400,84
Therapy	262	429	551,044	Causes	416	602	332,087	Reading	274	292	594,822	Anti-diabetes	86	91	333,531
Research	218	397	375,07	Serious	324	435	318,831	To neglect	275	296	585,785	Daily	85	95	303,24

Sentiment analysis of tweets can be useful in clinical settings as it increases our compression of user’s engagement on social networks and can help to improve strategic public health management.

**Figure 4:**
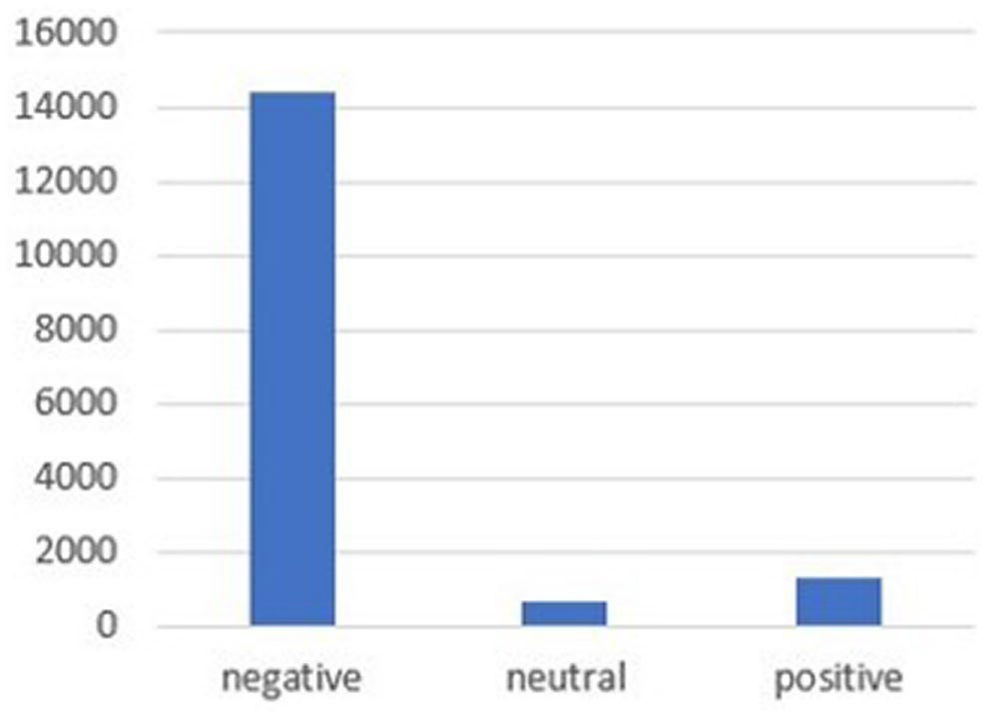
Sentiment analysis.

The combination of the Bert and VADER models produces 73.2% accuracy on the analyzed tweets for a total of 16,382 tweets with the following two types of output: the former differentiates 3 sentiments (positive, negative, and neutral), while the latter subdivides four basic emotions (anger, fear, joy, and sadness). As shown in Figure 4, tweets about diabetes and obesity in the health and disease framework are identified as communicating negative feelings at 87.5%, approximately 8% for positive feelings, and 4% for neutral feelings.

## 4. Discussion

Starting with the interpretation of the words in the factor axes in Table 2, an attempt is made to capture the most general cultural aspects that emerge from this study to identify the characterizing dimensions within which the issues carried by the clusters are located. Along the first dimension (Factor 1 with 44.09%) of the factorial space, the issue related to *Research* is played out. In fact, in its positive polarity, it seems to gather terms such as “study,” “NRP,” and “telemedicine.” In the negative polarity, the words, and particularly the verbs (increase, understand, and neglect) in common with fat/diet/overweight, recall the consequences of the two diseases. We could then hypothesize that this axis distinguishes the narratives produced because of how the different actors involved in the narrative (from healthcare institutions or people) relate to the theme of seeking treatment by linking diabetes and obesity to the necessary issue of going decisively reducing obesity and overweight. The second factor with 30.49% inertia, renamed as *Pathology,* seems to suggest an *a priori*-defined dimension that identifies the connection between the two diseases and their psychological sphere about them. In this sense, if on the positive pole, we find specific terms related to diabetes and obesity such as “mellitus,” “hypertension,” “eating,” and “sugar,” the negative polarity seems to express a specific reading of the patient’s psychological context. The third factor, renamed as *Prevention,* seems to decline the categorization of prevention and information as the main dimensions, which leads the Twitter audience to confront the need to relate to it. If committees and support associations are found on the positive pole, the negative polarity seems to highlight interest in different therapies and prevention. Within this cultural space, the representatives of four different perspectives of making sense of the function played by the network belong to the topics of diabetes and obesity.

From the clustering of the elementary contexts produced by the text fragments of the considered tweets, the narrative and representation of diabetes and obesity in CLUSTER 2 emerge as preponderant in the aspects of low focus on the research question and high characterization of the pathology. The cluster itself is renamed as *dangerous pathology* with reference to the society theory of risk and danger (Beck, 1992) and the distinction between danger, risk, and threats (Luhmann, 1993).

In this cluster, with greater weight in Factor 2, renamed as Pathology, the causes and complications of diabetes and obesity seem to be highlighted. Among the characterizing words, we find cardiovascular, severe, problems, and hypertension precisely highlighting the link between the two diseases with the increase and prevalence of cardiovascular disease. Interesting for interpretive purposes are the distributions of the keywords “death” and “dying” alongside “vaccinate” and “COVID,” ready to present what is an increased susceptibility to the development of infections for people with altered immune systems. In CLUSTER 3, it follows a representation of diabetes and obesity that bypasses issues such as pathology and research, focusing rather on nutrition and lifestyle issues, which we have renamed as a *healthy lifestyle*. The plans show that there are few elementary contexts devoted to prevention in this cluster; however, it focuses on both proper nutrition and dimensions related to psychological and emotional wellbeing. The lexical patterns include a careful assessment of food discourse with words such as pear, meat, fruit, and food, and at the same time highlight the correlation with mental health status (with the presence of words such as psychotherapist, psychiatrist, and psychoanalytic). Much of the literature has explored emotions, mental health status, and the presence of psychiatric conditions in relation to the risk of diabetes and obesity. Some scientific studies have highlighted the correlation between depressive symptoms and poorer levels of diabetes self-management, a significant correlation, especially in children and adolescents (Gonzalez et al., 2008). The diseases examined involve profound changes in many aspects of daily life, from eating habits to social relationships. There is evidence that emotional wellbeing is the domain of functioning in which diabetes affects and interferes most negatively, second only to physical health status (Nicolucci et al., 2013).

In contrast, CLUSTER 4 demonstrates elementary contexts with a moderate pathology component but involving topics devoted to research, which we have renamed as *medicine, institutions, and society,* and is the one where more references to the prevention dimension emerge (in fact, it has a greater weight on Factor 1 Research and Factor 3 Prevention).

Table 4 shows the construction of the set of words highlighting 14 November as World Diabetes Day. Thus, the lexical patterns in this cluster show the central role being played by information launchers, and the set of characterizing lemmas confirms how online information has potential impacts on the health of people with diabetes and obesity. At the same time, the importance of associationism as a contribution to the protection and improvement of health status is emphasized. A social commitment that should not be underestimated and that, as also demonstrated by the presence of the *Invisible Patients ONLUS Committee*, is also catapulted into the online world. Finally, CLUSTER 1 groups elementary contexts with a low pathology component but is also much more focused on the research dimension, coming very close to CLUSTER 4 even with reference to prevention. Renamed as *research and innovation*, the central element in CLUSTER 1 is the NRRP^5^ (National Recovery and Resilience Plan or Recovery Plan) along with words such as “region” and “innovation.”

The COVID-19 pandemic brings out the different weaknesses in the care and health system, with not a few differences between regions in northern, central, and southern Italy. The NRP, with Mission 6 of the National Recovery and Resilience Plan, allocates 15.63 billion euros for reform to define a new institutional set-up for prevention in health, environment, and climate. All this also represents a political and cultural response that can propose a paradigm shift in the management of people with diabetes and obesity. This dimension can also describe a change of perspective so that access to the different regional territories in Italy is equitable and uniform. A perspective (framed by words such as innovation, wellness, and care) that provides for the renewal of measures to ensure a better capacity to deliver and monitor care through more effective and faster modalities can be added to this.

**Figure 5:**
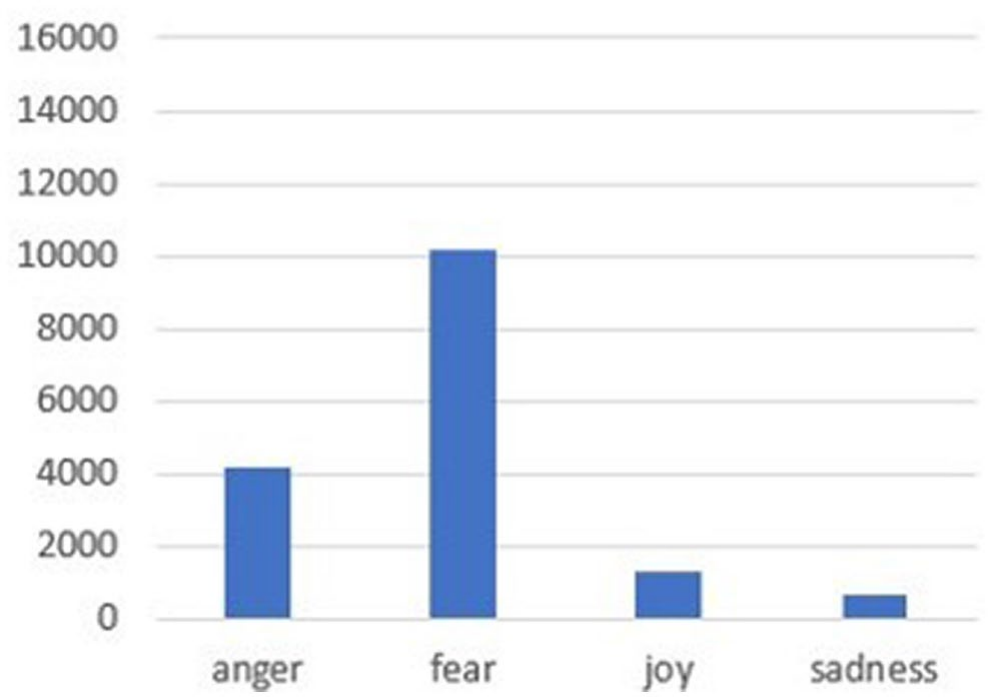
Sentiment analysis for four basic emotions.

The results can also be commented on by observing sentiment analysis output. Several studies show that many tweets related to health and particularly to the many morbidity issues contain a message that can be perceived as negative (Yu et al., 2022). This can be found in the narrative of the ebola virus or mental disorders (Oyeyemi et al., 2014; Wynn et al., 2017) and is even more confirmed in the narrative of the relationship between diabetes and obesity. In some cases, the content of the negative sentiment can also be explained by improper use of the terms diabetes and obesity, for example, by its inclusion in a provocation that, while inappropriate, turns out to be “ironic” (since the model used fails to read irony) or in a political squabble (“the only question an obese diabetic with heart disease who survives COVID-19 as a vaccinated person should ask is if I had not been vaccinated, I would be here tweeting”). Thus, Figure 5 shows that negative feelings are most represented by fear at 62% (also often related to the possibility of COVID-19 infection), anger at 25.8%, and sadness at 4.2%. In contrast, the tweets with positive sentiment tend to be closer to topics concerning healthy lifestyles, in fact, in tweets such as “sports and health extraordinary pair to prevent and cure chronic diseases depression anxiety obesity diabetes,” “the possibility of doing sports is useful for wellness and to prevent diabetes and obesity in young people,” and “diabetes overweight obesity (…) main benefits with fructose rich breakfast.” The totality of positive sentiments is found in those of the sentiment “joy,” represented by 8% in Figure 5, which shows among the top results in the table tweets about research and innovation such as “Useful protein discovered that links obesity to diabetes risk” or “From adolescence to adulthood obesity may play a role in the onset of diabetes (…) Petrelli’s therapy.” So, in many of the tweets where the message tends to have a positive sentiment, and consequently with those tagged as “joy,” the association with encouraging healthy behaviors is proposed by providing examples of how one can improve the quality of life by following some tips on nutrition and physical activity.

## 5. Conclusion

Today’s society has been transformed by the digital revolution through new communication devices, the enormous amount of data, data storage, and many other advances that also follow major changes in the dynamics and distribution of health and disease. Today, health is identified and recognized as a mirror of the state of wellbeing of an individual and society. This recognition has matured slowly over time, following particularly important stages. The definition of health itself is difficult to crystallize into a single sense that can be equally effective in different social, economic, and political contexts. In everyday life, people also attach quite different meanings to this word depending on personal characteristics (Blaxter, 1990). Thus, definitions of health and illness change over time in parallel with changes and advances in knowledge.

Social networks, such as Twitter, emerge as valuable resources for acquiring real-time data on a health topic or for leveraging platforms for scientific communication by experts. Today, there is a need to recognize the most discussed topics related to health and disease, as different discussions on social networks can influence patients’ opinions and behaviors.

In this study, we have analyzed a dataset of more than 20,000 tweets and shown that the language used to simultaneously discuss diabetes and obesity is variable and complex. Specifically, systematic evaluation on Twitter exploring keywords suggests several dimensions read in a correlation of interpretations. The first one is close to research, innovation, and the future. The second one is close to pathology and side effects. The third one has a dimension that includes mental and physical wellbeing and aims to promote healthy lifestyles and behaviors. Finally, a trend that deserves further investigation is the scope of a social dimension perhaps closer to a demand for receiving social support.

In our study, then, there is no dominant conversation thread, but there are several. The Italian tweets show how talking about diabetes and obesity in the context of health and illness does not mean just referring to the only “health” conditions; delving deeper, we discover dimensions closely related to each other and with a multiplicity of influencing factors.

Even more emphasized in the concept of health, understood as “a state of complete physical, mental and social wellbeing and not merely consisting of an absence of disease or infirmity,” is the emergence of both “individual factors” related to the body and psyche and “context factors,” since health is determined as a condition from the social context in which the individual is placed and interacts, and even the narrative of diabetes and obesity on Twitter frames a state of wellbeing considered as a whole.

The limitations of this study can be found in the lack of extension of this dataset. Moreover, with a view to future in-depth studies, we focus on demographic data capable of representing specific geographic locations in Italy in a way that differentiates the North, South, and Central regions.

However, the dimensions produced through the content analysis and the sentiment analysis allow not only represent the complexity of the representation of concepts such as diabetes and obesity for a social media community, but they are also useful to increase knowledge of how virtual platforms impact vulnerable populations. The presented study suggests the importance of implementing tools to rethink and better customize communication strategies for greater effectiveness of public health policies on complex pathologies such as the two considered.

## Data availability statement

The original contributions presented in the study are included in the article/supplementary material, further inquiries can be directed to the corresponding author.

## Ethics statement

Ethical approval was not required for the study involving human data in accordance with the local legislation and institutional requirements. The social media data was accessed and analyzed using Twitter API.

## Author contributions

FRL and FI contributed to the conceptualization, investigation and writing of the original draft. FRL contributed to conceptualization, investigation, data analysis and interpretation, to manuscript and literature review. FI contributed to methodology, formal analysis and data curation. All authors read and accepted the published version of the manuscript.

## Funding

This study was funded by Ministry of University and Research Grant PRIN 2020NCKXBR.

## Conflict of interest

The authors declare that the research was conducted in the absence of any commercial or financial relationships that could be construed as a potential conflict of interest.

## Publisher’s note

All claims expressed in this article are solely those of the authors and do not necessarily represent those of their affiliated organizations, or those of the publisher, the editors and the reviewers. Any product that may be evaluated in this article, or claim that may be made by its manufacturer, is not guaranteed or endorsed by the publisher.

## Footnotes

^1^Social Determinants of Health Report of a Regional Consultation Colombo, Sri Lanka, 2–4 October 2007.

^2^Health topics social determinants of health. Available at: https://www.who.int/health-topics/social-determinants-of-health#tab=tab_1 – Last accessed on: Jan 10^th^, 2023.

^3^Who, A Glossary Of Terms For Community Health Care And Services For Older Persons, 2004.

^4^The Passi (Progressi delle Aziende Sanitarie per la Salute in Italia) surveillance is characterized as a public health surveillance that collects, continuously and through sample surveys, information from the Italian adult population, aged 18–69 years, on lifestyles and behavioral risk factors related to the onset of chronic noncommunicable diseases, the degree of knowledge and adherence to intervention programs that the country is implementing for their prevention.

^5^The National Recovery and Resilience Plan, or Recovery Plan (NRRP), is the plan approved in 2021 by Italy to revive its economy after the COVID-19 virus pandemic. It is part of the European Union’s program known as Next Generation EU, a 750 billion euro European recovery fund.
